# Drivers of canine distemper virus exposure in dogs at a wildlife interface in Janos, Mexico

**DOI:** 10.1002/vro2.7

**Published:** 2021-05-05

**Authors:** Rocío Almuna, Andrés M. López‐Pérez, Rosa E. Sarmiento, Gerardo Suzán

**Affiliations:** ^1^ ECOS (Ecosystem–Complexity–Society) Laboratory, Center for Local Development (CEDEL), Villarrica Campus Pontificia Universidad Católica de Chile Villarrica Chile; ^2^ Centro Regional de Investigación para la Sostenibilidad de la Agricultura y los Territorios Rurales_Ceres Pontificia Universidad Católica de Valparaíso Valparaíso Chile; ^3^ Department of Veterinary Medicine and Epidemiology University of California California USA; ^4^ Departamento de Etología, Fauna Silvestre y Animales de Laboratorio, Facultad de Medicina Veterinaria y Zootecnia Universidad Nacional Autónoma de México, Ciudad Universitaria Mexico City México; ^5^ Departamento de Microbiología e Inmunología, Facultad de Medicina Veterinaria y Zootecnia, Universidad Nacional Autónoma de México, Ciudad Universitaria Mexico City México

**Keywords:** canine distemper virus, disease spillover, domestic dog, domestic‐wildlife interface, wild carnivores

## Abstract

**Background:**

Human population expansion has increased the contact between domestic animals and wildlife, thereby increasing the transmission of infectious diseases including canine distemper virus (CDV). Here, we investigated the risk factors associated with CDV exposure in domestic and wild carnivores from the Janos Biosphere Reserve (JBR), Mexico.

**Methods:**

A cross‐sectional household questionnaire study was performed in four rural towns to investigate the risk factors associated with the presence of CDV in domestic and wild carnivores from the JBR, Mexico. In addition, we tested serum samples from 70 dogs and three wild carnivores, including one bobcat (Lynx rufus), one striped skunk (*Mephitis mephitis)* and one gray fox (*Urocyon cinereoargenteus*) for CDV antibodies using immunochromatographic and viral neutralization assays.

**Results:**

Overall, 62% of domestic dogs were seropositive for CDV, and the presence of antibodies was significantly higher in free‐roaming owned dogs than dogs with restricted movement. Among the wild carnivores, only the bobcat was seropositive. The rate of vaccination against CDV in dogs was low (7%), and there was a high rate of direct interactions between domestic dogs and wild carnivores.

**Conclusion:**

Our serological assays show that CDV is circulating in both domestic dogs and wild carnivores, suggesting cross‐species transmission. Our finding of low vaccination rates, high number of unrestrained owned dogs and direct interactions between wildlife and domestic animals reported in the region may be perpetuating the high prevalence of the virus and increasing the risk of CDV transmission between wild and domestic carnivores. Therefore, long‐term longitudinal studies are recommended in order to monitor infectious diseases at the domestic‐wildlife interface in this highly biodiverse region.

## INTRODUCTION

Over the past few years, the accelerated growth and expansion of humans and domestic dogs (*Canis lupus familiaris*) have impacted ecosystem structure and function, increasing direct and indirect interactions with wildlife. Often, these interactions have been the starting point for the spread of serious diseases that affect animals of different taxa; canine distemper virus (CDV) is an example of this phenomenon. This pathogen can cause high mortality rates, representing a major welfare and conservation concern worldwide for domestic dogs and wild carnivores.^1^, ^2^ The pathogen is a large, single‐stranded RNA virus belonging to the genus *Morbillivirus* in the family *Paramyxoviridae* that causes a highly infectious and severe systemic disease affecting domestic and wild carnivores worldwide.^3^, ^4^ CDV has been associated with population declines of wild carnivore species, such as the black‐footed ferrets (*Mustela nigripes*),^5^ African lions (*Panthera leo*),^6^ and African wild dogs (*Lycaon pictus*),^7^, ^8^ and some of these outbreaks have been directly associated with the spillover of CDV from domestic dogs.^6^, ^9^, ^10^

In Mexico, CDV is a common pathogen in household dogs,^11^, ^12^ but little is known about its eco‐epidemiology in free‐roaming dogs in rural areas where wild and domestic interface occurs. To our knowledge only two studies have been conducted on domestic and wild carnivores in Mexico. One of them was a serological survey of CDV in jaguars and domestic dogs in the state of Campeche in tropical Mexico, and the other included both molecular and serological surveillance in both domestic and wild carnivores in the state of Chihuahua in northern Mexico.^13^, ^14^

The Janos Biosphere Reserve (JBR) is located in northwestern Mexico and is considered a priority conservation site for North American biodiversity.^15^, ^16^ Carnivores are the second most represented Order at JBR, with a total of 12 species inhabiting the area, including the Mexican gray wolf (*Canis lupus baileyi*) which is an endangered species.^17^, ^18^ The black‐footed ferret is an endangered mustelid that suffered a dramatic decline explained by the decline of its main prey the prairie dog (*Cynomis spp*.), as well as by CDV infection.^19^ The black‐footed ferret was re‐introduced in JBR in 2001, but unfortunately there are no current records indicating that the reintroduced populations have thrived.^14^ Infections such as CDV may have constrained reintroduction success. Rural human settlements and agriculture activities are increasing in this region,^20^ shrinking native grasslands, which in turn might increase contact rates between domestic and wildlife and modify pathogen dynamics.

It is important to note that dogs have played important roles in human history as companion animals, household guardians and livestock protectors.^21^ Dog populations have reached over 1.2 billion individuals globally in urban, rural and natural protected areas, increasing ecological and epidemiological concerns for animal and public health.^3^ Sometimes, dogs are unsupervised and have no spatial restriction by their owners; these are known as free‐roaming dogs. Free‐roaming dogs are known to be an impediment to wildlife conservation goals.^22^ These dogs have been responsible for introducing infectious diseases whose causal agents have adapted to new host species around the world, including rabies, canine parvovirus and CDV.^23^, ^24^ Therefore, studying the ecology of free‐roaming dogs and the epidemiology of critical infectious diseases affecting wild carnivores, such as CDV, is crucial for designing specific conservation strategies to reduce the impact of domestic dog diseases on native wildlife.

The goals of this study were to [1] determine the presence or absence of antibody response against CDV in domestic dogs and wild carnivores, [2] assess domestic and wild carnivore interactions and [3] identify risk factors for exposure to CDV in domestic dogs in the JBR based on epidemiological and ownership patterns.

## MATERIALS AND METHODS

### Study area

**FIGURE 1:**
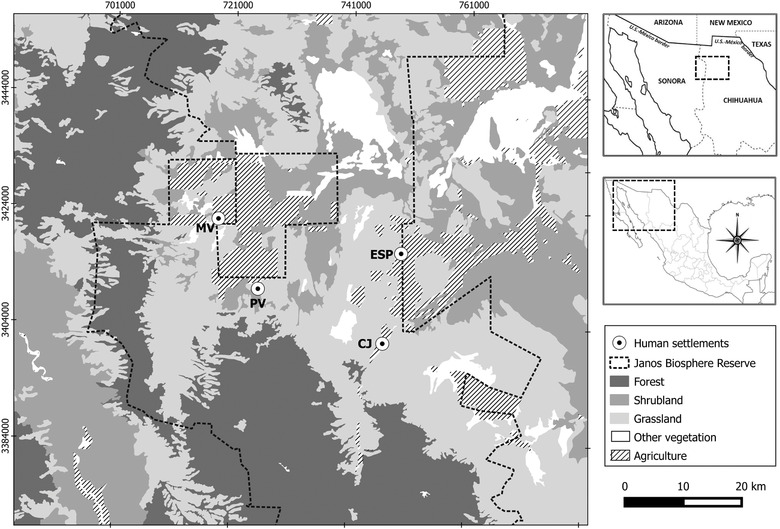
Sampling cites along the Janos Biosphere Reserve, Chihuahua, México. Capital letter refer to locations (MV: Monte Verde; PV: Pancho Villa; ESP: Ejido San Pedro; CJ: Casa de Janos)

The JBR (31°11′7.63″ – 30°11′27.45″ N and 108°56′49.17″ – 108°56′22.1″ O) covers most of the Municipality of Janos, Chihuahua with an area of 530,000 ha (Figure 1). It shares a border with the United States and the state of Sonora, Mexico. There are 53 rural towns within the reserve, with a population of less than 2500 inhabitants (Semarnat, 2013).^25^ The JBR is located within the Chihuahuan Desert ecoregion at elevations from 1200 to 2700 meters altitude. The main types of vegetative cover are grasslands, shrubs and forests, and it is inhabited by 79 species of mammals.^16^

### Sampling design

We conducted the study during March 2015 in four towns (Ejido San Pedro [ESP], Monte Verde [MV], Pancho Villa [PV] and Casa de Janos [CJ]) (Figure 1). To assess ownership patterns and domestic and wild carnivore interactions, we conducted a cross‐sectional household questionnaire. We randomly selected 4–6 blocks from each town, each block had a perimeter of approximate 1000 mts. The questionnaires were deployed at each block by choosing a random house and asking if the inhabitants owned a dog and consented to participate. If consent was not provided or the house did not own a dog, we continued around the block until obtaining the consent of a dog‐owning household. To determine the sample size to evaluate the prevalence of antibodies against CDV in the study area, we estimated the number and densities of domestic dogs at each village by counting sighted dogs along linear transects 100 meters wide. The length of the transects varied depending on the size of the towns (ESP 3.5 km, MV 11.7 km, CJ 4 km and PV 8.3 km). The surface area of each town was calculated by drawing a perimeter connecting the most external households in each town using Google Earth V 7.1.4.1529 satellite imagery (Maxar technologies) from 2014. This gave us an estimated population size of 306 dogs in total for all four towns. The target sample size was calculated based on an estimated 72% prevalence of antibodies against CDV as reported in similar studies conducted with dogs in Chile and India^9^, ^26^ and a 95% confidence interval at 10% absolute precision. This yielded a target sample size of 68 dogs. We sampled 70 dogs (20 in ESP, 20 in MV, 10 in PV and 20 in CJ) to assess CDV exposure and acquired information about those dogs and their ownership features using a questionnaire answered by their owners. Questions were related to dog's general information and care, such as age, diet, function performed at home (companion animal, guard dog to protect the household, shepherd dog to protect the livestock), deworming, vaccination status, and whether the dogs were restricted in their movements or free‐roaming. Free‐roaming was defined as dogs allowed to roam freely on the streets and natural area. In addition, we asked other questions about interactions between wild carnivores and domestic dogs (e.g. predation and habitat overlap) in the towns and surrounding areas. To assess CDV serology in the study population of domestic dogs, a blood sample was collected from the cephalic vein of each dog with prior authorization from the owners. The study was approved and performed based on UNAM Veterinary School's animal care and use protocol and guidelines (03‐V‐89).

### Capture of wild carnivores

Wild carnivores were captured near Casa de Janos along a transect of 10 trapping stations placed along roads and trails spaced 500–800 meters apart (spanning a total of seven kilometers in length). Each station consisted of a box trap (30″ × 30″ × 70″ or 60″ × 20″ × 28″, Tomahawk Live Trap Inc., WI, USA), and a leg‐hold soft catch trap (#1.75 or #3 Victor Coil Soft Catch, Cleveland, OH, USA), spaced 20–50 meters apart. Each station was baited with sardine, tuna and commercial lure (Kishel's, East Aurora, NY). Traps were active at night from 14th March to 22nd March resulting in a sampling effort of 90 trap nights, checked twice per night.

We captured three wild carnivores: a gray fox (*Urocyon cinereargenteus*), a bobcat (*Lynx rufus*) and a striped skunk (*Mephitis mephitis*); each of these individuals was sampled. These individuals were immobilised with a mixture of ketamine hydrochloride (Anesket; Pisa, Atitalaquia, Hidalgo, Mexico) and xylazine hydrochloride (Rompun; Pisa, Atitalaquia, Hidalgo, Mexico) according to the doses described elsewhere.^27^ The doses used for each individual can be found in Table 1. All procedures for trapping and handling carnivores followed the guidelines of the American Society of Mammologists^28^ and were approved by the Mexican Secretary of Environment and Natural Resources (Permit FAUT‐0250).

**TABLE 1:** 

Species	Immobilization agent dose	Sex	Sign of disease	Antibodies against CDV
Grey fox *Urocyon cinereargentus*	Ketamine 20 mg/kg Xylazine 1 mg/kg	Male	None	No
Bobcat *Lynx rufus*	Ketamine 10 mg/kg Xylazine 1.5 mg/kg	Female	None	Yes
Hooded skunk *Mephitis macroura*	Ketamine 15 mg/kg Xylazine 8 mg/kg	Male	None	No

### Sample collection and storage

Both domestic dogs and wild carnivores were weighed, sexed, identified to species level, and examined for any sign of disease. Blood samples were collected (3 mL each) in Vacutainer tubes without anticoagulant from the cephalic or femoral vein; tubes were centrifuged at 3000 RPM for 15 min to obtain serum. Serum samples were transferred into 1.5 ml cryotubes and subsequently stored at −4°C. Samples were later transferred to a −70°C freezer until processed.

### Laboratory analysis procedure

Domestic dog serum samples were analysed using a rapid diagnostic kit for immunochromatography (BioNote Inc., Zapopan, Jalisco, México) with a sensitivity of 98.6% and specificity of 100%, following the manufacturer's instructions. Wild carnivore samples were analysed by viral infectivity neutralization, using Crandell‐Rees Feline Kidney Cell cell culture line (CRFK ATCC CCL‐94) and attenuated Bussell strain virus based on the procedure previously described by Nakano et al.^29^ Positive samples were those in which there was a loss of the infecting capacity of the virus due to the reaction of the protective antibodies present in the sample. Neutralizing antibody titer was determined by calculating the level of the antibodies at the greatest dilution (lowest concentration) of the serum sample at which the assay still produced a detectable positive result.

### Data analysis

The association between the presence of antibodies in dogs (binary response variable) and the dog's locality, function at home, number of dogs per household, free‐roaming and age (explanatory variables) was assessed using a logistic regression model using SPSS software. We assessed collinearity using a Spearman rank‐order correlation (rho, ρ) to exclude strongly correlated variables (rs > 0,6; *p* < 0,05) from the model. Prior to multiple logistic regression, we performed simple logistic regression analyses between each of the explanatory variables and the dependent variable. In the multiple models, we only included variables that had a significant association (*p* < 0.05) with the dependent variable. We also used the Spearman correlation coefficient for non‐parametric data to evaluate the relationship between the presence of antibodies in domestic dogs, average age and dog densities per town.

## RESULTS

Of the 70 domestic dogs sampled, five individuals were excluded from the analysis because they had been vaccinated against CDV (*n* = 65). Overall, antibodies were detected in 62.5%, (95% CI [59.8, 65.1]), and specific values from each town were 47% in ESP, 63% in CJ and 70% in PV and MV. Nine dogs had clinical signs associated with distemper (weakness, lack of appetite, malnutrition, vomiting, diarrhea, neurological alterations, nasal secretions, conjunctival secretions, cough or sneezing). Six of these were also seropositive for IgG against CDV 67% (6/9). Out of the three wild carnivores sampled, a female bobcat was the only positive individual with a high titer of neutralizing antibodies (1:15,849).

Seventy‐four percent (52/70) of the households interviewed claimed to have seen free‐roaming owned and stray dogs (unowned) on the streets in the towns and/or in the protected native grasslands. During the survey we were able to confirm the presence of stray dogs in two of the four towns. Using our demographic data, we estimated a dog population density between 0.5–1.8 dogs/ha (Table 2). The majority of the dogs were males (64%). The average age was 2.8 years, and 63% of the individuals ranged from 1 to 5 years old. The majority of owners had one or two dogs in their households (67%), and veterinary attention (preventive or for treatment) was very low (7%). Mostly, dogs were mongrels and fulfilled the functions of companion animal (64%), guard dog (27%) and shepherd dog (9%).

**TABLE 2:** Dog's calculated demographic data in the sampled localities

	Ejido San Pedro	Monte Verde	Casa de Janos	Pancho Villa
Dogs counted	64 dogs in 35 ha	134 dogs in 117 ha	54 dogs in 40 ha	54 dogs in 102 ha
Dogs calculated densities	1.8 dogs/ha	1.1 dogs/ha	1.4 dogs/ha	0.5 dogs/ha

The questionnaire results regarding ownership patterns indicated that the majority of owned dogs were free‐roaming in three of the four towns (MV 65%, PV 80%, CJ 65%), whereas less than half of the owned dogs were free‐roaming in ESP (48%). Eighty percent of shepherd dogs and 70% of guard dogs were allowed to roam freely in the community and accompanied their owners to the cultivated lands immersed in the protected area.

According to the questionnaire results, interactions between free‐roaming dogs and wild carnivores were mentioned in all locations. PV and MV were the towns with the highest percentage of recognition of interactions (40% and 40%, respectively), followed by CJ (35%) and ESP (25%). Direct contacts between free‐roaming dogs and coyotes (*Canis latrans*), skunks (*Mephitis* spp.) and bobcats were described, stating that free‐roaming dogs were usually seen alone or in packs wandering into the protected area. Coyote, bobcat, skunk and gray fox were the most frequently seen species near human settlements. The highest number of wildlife sightings was reported for CJ, including a recording of a black bear (*Ursus americanus*), while the lowest sighting was in ESP.

The logistic regression model (Table 3) revealed that free‐roaming is a risk factor for CDV exposure (OR = 3.05, *p* < 0.05) and we found no association between the function of the dog (guardian, shepherd, companion) and CDV seropositivity.

**TABLE 3:** Logistic regression model for estimating the risk factors for the exposure to canine distemper virus in domestic dogs in the Janos Biosphere Reserve, Mexico

Predictive parameter	N (%)	Coefficient	OR	95% CI	*p* value
Age (continuous)		0.23	1.26	0.98–1.61	0.07
Free‐roaming					
Free‐roaming dogs	39 (60%)	1.11	3.05	1.04–8.96	0.04*
Dog per house		−0.43	0.65	0.41–1.02	0.06
Function					
Guardian dog	45 (64%)	1.4	4.06	0.97–16.55	0.06
Shepherd dog	19 (27%)	0.27	1.3	0.14–7.07	0.99
Companion animal	6 (9%)	0.14	1.15	0.85–4.16	0.12

* Significant values.

On the other hand, based on the Spearman correlation analysis, we found a significant negative correlation between the presence of antibodies against CDV and dog densities (*r* [63] = −0.95; *p* < 0.05) and a positive correlation between the antibodies and average ages (*r* [63] = 0.95; *p* < 0.05). Thus, the presence of antibodies decreases as dog population density increases, and older dogs are more likely to have been exposed to CDV.

## DISCUSSION

Our study on the risk factors for CDV exposure in domestic dogs shows that this infectious agent is circulating in both dogs and wild carnivores and that free‐roaming dogs have an increased risk of exposure. Morbilliviruses are a growing epidemiological problem because of their ability to infect multiple species and have been associated with mass mortality in marine and terrestrial mammals, including several species of wild carnivores worldwide.^4^, ^30^ It affects the body systemically, with generalized signs such as fever, vomiting and diarrhea, along with skin disorders and neurological signs in advanced stages.^31^ It is a major welfare concern in dogs, but overall vaccination rates are low.^32^ The spillover of CDV from domestic dogs to wild carnivores has been documented worldwide and has a level impact that varies among different native carnivore populations. There are a few cases where it has even brought populations to the brink of local extinction, as it is the case of the black‐footed ferret in North America^5^ and African wild dogs in the Serengeti.^7^

The ecology and evolution, host range and spillover events of CDV have been scarcely described worldwide, and little is known about its impact on wildlife from Mexico. Our results offer valuable insight into the relationships between dog demography and ecology and CDV exposure in a human‐domestic‐wild interface in northwestern Mexico. The high prevalence of antibodies against CDV in dogs (62%) was consistent with several reports worldwide. For instance, antibodies against CDV in domestic dogs from rural areas in northern Chile ranged from 72 to 75%,^9^ and in India, Belsare and Gompper^26^ found a similar prevalence of over 72% in rural free‐roaming dogs. In rural areas of Southern Chile, there was a high overall seroprevalence of CDV in (52%), which ranged from 36% to 80% depending on the site.^33^ In Mexico, a 52% seroprevalence has been reported in free‐roaming dogs from the Calakmul Biosphere Reserve.^13^ All these studies suggest that free‐roaming dogs may play a role in increasing the prevalence of the virus in wildlife, although the possibility of wildlife transmitting CDV to dogs cannot be ruled out. CDV can be spread through feces, urine and other body secretions, so it is easily transmitted between individuals inhabiting the same territory,^4^ even if there is no direct contact. If unvaccinated dogs roam freely, the virus can widely disseminate not only between dogs, but also among other carnivores. The results of this research, along with the low vaccination rate against CDV, highlight the need to devote special attention to responsible ownership practices, including supervision and veterinary care, to help control spread of this infectious disease.^9^

Enforcing widespread dog vaccination can reduce CDV infection among dogs^34^ and could therefore prevent its transmission to wild carnivores. However, further research is needed to assess the effectiveness of this management option to protect species‐rich ecosystems from CDV. Besides the high proportion of unvaccinated dogs (93%, 65/70), the population density has been in many studies a plausible explanation for the high CDV antibody prevalence. However, contrary with several studies which reported that CDV prevalence is often positively associated with population density,^6^, ^35^ we found a lower prevalence of CDV seropositivity in dogs in places with higher dog density. Our results may reflect a decrease of the direct contact between individuals or infected feces due to the higher rate of dogs with restricted movement. Another likely explanation for this result could be a previous decrease of dog population driven by a high mortality rate caused by CDV outbreaks. Nevertheless, although CDV is generally believed to be a density‐dependent disease, several other studies have surprisingly found the virus can persist among low‐density host populations.^6^, ^36^ San Pedro was the site with the lowest percentage of free‐roaming dogs, while Pancho Villa had the highest. This is consistent with the results of low presence of antibodies in San Pedro, and the high presence of antibodies in Pancho Villa. This finding, along with the results from the logistic regression analysis showed that free‐roaming is a risk factor for the presence of antibodies against the virus, while the densities did not show any association in the logistic regression. In our study, we only included owned domestic dogs, but we consider that that stray (unowned) dogs could also have an impact in CDV epidemiological dynamics. Thus, further studies are needed to understand the role of stray dogs in the transmission of CDV in JBR.

A bobcat captured 5 km away from Casa de Janos was the only wild carnivore presenting antibodies against CDV. A previous study conducted in JBR reported the presence of CDV in eight wild carnivores through molecular analysis, including bobcats captured near Casa de Janos and Pancho Villa.^16^ Similarly, Harrison in 2010 reported positive results in bobcats through serology in the Chihuahua desert of New Mexico.^37^ Likewise, in eastern Canada, Daoust et al^38^ found one bobcat and six lynx (*Lynx canadensis*) that died from CDV, as demonstrated by serology and molecular analysis. A number of people in this study reported direct interactions between domestic dogs and wild carnivores. It is therefore important to determine whether domestic animals are playing a role in the transmission of the virus. Infected animals, either dogs or other carnivores, can be reservoirs of the virus^39^ and transmit it to other species that do not usually have contact with human or domestic dog populations. Due to the lack of historic exposure to CDV, other previously unexposed populations could be highly susceptible to the virus and could be strongly affected.^40^

The study area is immersed in one of the most important reserves for the conservation of mammals in Mexico. The results of this study, together with those reported by Moreno^14^ indicate that the virus is circulating in carnivores in the region; however there is no information on the potential negative impact on the populations of wild carnivores in the JBR. Considering the ongoing attempts to re‐introduce the black‐footed ferret to this region^16^ and the high susceptibility that this species has historically shown to the virus,^40^ better understanding of the epidemiological characteristics of the CDV is especially important in this area.

Controlling or eradicating canine distemper is a challenging and complex goal since the virus circulates within multiple hosts within ecosystems, and its means of transmission and pathogenesis are evolving.^41^ Even though this study does not demonstrate direct interspecific infection risk for CDV between domestic and wild populations, spill‐over cannot be ruled out. Therefore, it is important to investigate the circulating strains of this virus and assess the need to prevent future outbreaks that could affect highly susceptible wild carnivore species.

## AUTHOR CONTRIBUTIONS

Experimental design: Rocío Almuna, Andrés M. López‐Pérez and Gerardo Suzán. Sampling: Rocío Almuna and Andrés M. López‐Pérez. Laboratory analysis: Rocío Almuna and Rosa E. Sarmiento. Data analysis: Rocío Almuna and Andrés M. López‐Pérez. Writing the manuscript: Rocío Almuna, Andrés M. López‐Pérez and Gerardo Suzán.

## References

[1] Daszak P , Cunningham AA , Hyatt AD . Emerging infectious diseases of wildlife. Biodiversity and human health. Sci Compass. 2000;287:443–9. 10.1126/science.287.5452.443 10642539

[2] Smith KF , Acevedo‐Whitehouse K , Pedersen AB , Acevedo K , Pedersen A . The role of infectious diseases in biological conservation. Anim Cons. 2009;12(1):1–12.

[3] Gompper ME . Free‐ranging dogs and wildlife conservation. New York: Oxford University Press; 2014.

[4] Deem SL , Spelman LH , Yates RA , Montali RJ . Canine distemper in terrestrial carnivores: a review. J Zoo Wildl Med. 2000;31(4):441–51. 11428391 10.1638/1042-7260(2000)031[0441:CDITCA]2.0.CO;2

[5] Belan J , Biggins D , Garelle D , Griebel RG , Hughes JP . Mustela nigripes. The IUCN red list of threatened species 2015. 10.2305/IUCN.UK.2015-4.RLTS.T14020A45200314.en. Accessed 6 February 2020.

[6] Cleaveland S , Appel MJGJ , Chalmers WSK , Chillingworth C , Kaare M , Dye C . Serological and demographic evidence for domestic dogs as a source of canine distemper virus infection for Serengeti wildlife. Vet Microbiol. 2000;72:217–27. 10727832 10.1016/s0378-1135(99)00207-2

[7] Goller KV , Fyumagwa, RD , Nikolin V , East, ML , Kilewo M , Speck S , et al. Fatal canine distemper infection in a pack of African wild dogs in the Serengeti ecosystem, Tanzania. Vet Microbio. 2010;146(3‐4):245–52. 10.1016/j.vetmic.2010.05.018 20684868

[8] Alexander KA , Appel MJG . African wild dogs (*Lycaon pictus*) endangered by a canine distemper epizootic among domestic dogs near the Masai Mara National Reserve, Kenya. J Wildl Dis. 1994;30:481–5. 7760475 10.7589/0090-3558-30.4.481

[9] Acosta‐Jamett G , Chalmers WSK , Cunningham AA , Cleaveland S , Handel IG , Bronsvoort BM de C . Urban domestic dog populations as a source of canine distemper virus for wild carnivores in the Coquimbo region of Chile. Vet Microbiol. 2011;152(3–4):247–57. 21641130 10.1016/j.vetmic.2011.05.008

[10] Marcacci M , Ancora M , Mangone I , Teodori L , Di Sabatino D , De Massis F , et al. Whole genome sequence analysis of the arctic‐lineage strain responsible for distemper in Italian wolves and dogs through a fast and robust next generation sequencing protocol. J Virol Methods. 2014;202:64–8. 24642239 10.1016/j.jviromet.2014.02.027

[11] Simon‐Martínez J , Ulloa‐Arvizu R , Soriano VE , Fajardo R . Identification of a genetic variant of canine distemper virus from clinical cases in two vaccinated dogs in Mexico. Vet J. 2008;175(3):423–6. 17382567 10.1016/j.tvjl.2007.01.015

[12] Gámiz‐Mejía CE , Simon‐Martinez J , Fajardo‐Muñoz RC . Identification of new genovariants of canine distemper virus in dogs from the State of Mexico by analyzing the nucleocapsid gene. Arch Med Vet. 2012;44:53–8.

[13] Ortiz SE . Investigación Epidemiológica del Virus del Distemper Canino en Perros Domésticos, Jaguares y Pumas en los alrededores de la Reserva de la Biósfera Calakmul en el Sur de México. Mexico City: Universidad Nacional Autónoma de México; 2014.

[14] Moreno K . Estudio serológico y molecular de distemper y parvovirus canino en comunidades de carnívoros de la reserva de la Biósfera de Janos, Chihuahua. 2016. Master's thesis. Facultad de Medicina Veterinaria. Universidad Nacional Autónoma de México. Retrieved from (http://132.248.67.65:8080/bitacoras/bitacoras.jsp?recurso=tesiunam&url=http%3A%2F%2F132.248.9.195%2Fptd2016%2Fagosto%2F0749121%2FIndex.html)

[15] Ceballos G , Pacheco J , List R , Manzano‐Fischer P , Santos G , Royo M . Prairie dogs, cattle, and crops: diversity and conservation of the grassland‐shrubland habitat mosaics in northwestern Chihuahua, Mexico. In: Cartron JLE , Ceballos G , Felger RS , editors. Biodiversity, ecosystems, and conservation in Northern Mexico. New York: Oxford University Press; 2005. p. 425–38.

[16] List R , Pacheco J , Ponce E , Sierra‐Corona R , Ceballos G , Sierra R , et al. The Janos Biosphere Reserve, Northern Mexico. Int J Wilderness. 2010;16(2):35–41.

[17] Lara‐Díaz NE , López‐González CA , Coronel‐Arellano CA , Cruz‐Romo JL . Nacidos libres: en el camino a la recuperación del lobo mexicano. CONABIO Biodiversitas. 2015;116:1–6.

[18] Pacheco J , Ceballos G , List R . Reintroducción del hurón de patas negras en las praderas de Janos, Chihuahua. CONABIO Biodiversitas. 2002;42:1–5.

[19] Biggins DE , Godbey JL , Vargas A , Anderson SH . Influence of prerelease experience on reintroduced black‐footed ferrets (*Mustela nigripes)* . Biol Conserv. 1999;89:121–9.

[20] Ceballos G , Davidson A , List R , Pacheco J , Manzano‐Fischer P , Santos‐Barrera G , et al. Rapid decline of a grassland system and its ecological and conservation implications. PLoS One. 2010;5(1):e8562. 20066035 10.1371/journal.pone.0008562 PMC2797390

[21] Hart, LA , Yamamoto, M . Dogs as helping partners and companions for humans. In: Serpell J , editor. The domestic dog. Cambridge: Cambridge University Press; 2010. p. 247–70.

[22] Young J. , Bergman D , Ono M . Bad dog: feral and free‐roaming dogs as agents of conflict. An Conservation. 2018;21(4):285–6.

[23] Atickem A , Bekele A , Williams SD . Competition between domestic dogs and Ethiopian wolf (*Canis simensis*) in the Bale Mountains National Park, Ethiopia. Afr J Ecol. 2010;48(2):401–7.

[24] Doherty TS , Dickman CR , Glen AS , Newsome TM , Nimmo DG , Ritchie EG , et al. The global impacts of domestic dogs on threatened vertebrates. Biol Conserv. 2017;210:56–9.

[25] Secretaría de Medio Ambiente y Recursos Naturales (Semarnat). Programa de Manejo Reserva de la Biósfera de Janos. 2013. https://www.rufford.org/files/Programa%20de%20Manejo%20Reserva%20de%20la%20Biosfera%20Janos.pdf. Accessed 12 November 2019.

[26] Belsare AV , Gompper ME . Assessing demographic and epidemiologic parameters of rural dog populations in India during mass vaccination campaigns. Prev Vet Med. 2013;111(1–2):139–46. 23664490 10.1016/j.prevetmed.2013.04.003

[27] Kreeger TJ , Raath JP , Arnemo JM . Handbook of wildlife chemical immobilization. Fort Collins, Colorado: Wildlife Pharmaceuticals Inc; 2002.

[28] Sikes RS , Gannon WL . Guidelines of the American Society of Mammologists for the use of wild mammals in research. J Mammal. 2011;92(1):235–53. 10.1093/jmammal/gyw078 PMC5909806 29692469

[29] Nakano H , Kameo Y , Andoh K , Ohno Y , Mochizuki M , Maeda K . Establishment of canine and feline cells expressing canine signaling lymphocyte activation molecule for canine distemper virus study. Vet Microbiol. 2009;133:179–83. 18687538 10.1016/j.vetmic.2008.06.016 PMC7125910

[30] Kuiken T , Kennedy S , Barrett T , Van De Bildt MWG , Borgsteede FH , Drew SD , et al. The 2000 canine distemper epidemic in caspian seals (*Phoca caspica*): pathology and analysis of contributory factors. Vet Pathol. 2006;43:321–38. 16672579 10.1354/vp.43-3-321

[31] Beineke A , Puff C , Seehuse F , Baumgärtner W . Pathogenesis and immunopathology of systemic and nervous canine distemper. Vet Inmun Immunopat. 2009; 127:1–18. 10.1016/j.vetimm.2008.09.023 19019458

[32] Void V . The impact of companion animal problems on society and the role of veterinarians. Vet Clinics North Am Small Anim Pract. 2009;39(2):327–45. 10.1016/j.cvsm.2008.10.014 19185196

[33] Acosta‐Jamett G , Surot D , Cortes M , Marambio V , Valenzuela C , Vallverdu A , et al. Epidemiology of canine distemper and canine parvovirus in domestic dogs in urban and rural areas of the Araucania region in Chile. Vet Microbiol. 2015;178(3–4):260–4. 26013417 10.1016/j.vetmic.2015.05.012

[34] Viana M. , Cleaveland, S . Matthiopoulos J , Halliday J , Packer C , Craft ME , et al. Dynamics of a morbillivirus at the domestic‐wildlife interface: Canine distemper virus in domestic dogs and lions. Proc Natl Acad Sci. 2015;112(5):1464–9. 25605919 10.1073/pnas.1411623112 PMC4321234

[35] Acosta‐Jamett G. , Cleaveland S. , Cunningham AA , Bronsvoort BM de C . Demography of domestic dogs in rural and urban areas of the Coquimbo region of Chile and implications for disease transmission. Prev Vet Med. 2010;94(3–4):272–81. 20096943 10.1016/j.prevetmed.2010.01.002

[36] Almberg ES , Cross PC , Smith DW . Persistence of canine distemper virus in the Greater Yellowstone Ecosystem's carnivore community. Ecol Appl. 2010;20(7):2058–74. 21049890 10.1890/09-1225.1

[37] Harrison RL . Ecological relationships of bobcats (*Lynx rufus*) in the Chihuahuan Desert of New Mexico. Southwest Nat. 2010;55(3):374–81.

[38] Daoust PY , McBurney SR , Godson DL , van de Bildt MWG , Albert DME , Osterhaus ADME . Canine distemper virus‐associated encephalitis in free‐living lynx (*Lynx canadensis*) and bobcats (*Lynx rufus*) of eastern Canada. J Wildl Dis. 2009;45(3):611–24. 19617471 10.7589/0090-3558-45.3.611

[39] Sepúlveda MA , Singer RS , Silva‐Rodriǵuez E , Eguren A , Stowhas P , Pelican K . Invasive American Mink: linking pathogen risk between domestic and endangered carnivores. EcoHealth. 2014;11:409–19. 24604545 10.1007/s10393-014-0917-z

[40] Williams ES , Thorne ET . Infectious and parasitic disease of captive carnivores, with special emphasis on the black‐footed ferret (*Mustela nigripes*). Rev Sci Tech Off Int Epiz. 1996;15(1):91–114. 10.20506/rst.15.1.915 8924718

[41] Nikolin VM , Olarte‐Castillo XA , Osterrieder N , Hofer H , Dubovi E , Mazzoni CJ , et al. Canine distemper virus in the Serengeti ecosystem: molecular adaptation to different carnivore species. Mol Ecol. 2016;26(7):2111–30. 27928865 10.1111/mec.13902 PMC7168383

